# Supplementary material for: Patterns of Intron Gain and Loss in Fungi
Source: PLoS Biol. 2004 Nov 30;2(12):e422. doi: 10.1371/journal.pbio.0020422 (PMC532390; doi:10.1371/journal.pbio.0020422)
Supplement: Table S1 — Also available at http://genes.mit.edu/NielsenEtAl/. (4.3 MB ZIP). [file pbio.0020422.st001.zip › NielsenEtAl/html/1242.html]

AN1761.1.NCU10046.1.MG00600.1.FG09313.1


```
 CLUSTAL W (1.82) Multiple Sequence Alignments - Introns Inserted


Sequence 1: MG00600.1	204 aa
Sequence 2: FG09313.1	211 aa
Sequence 3: NCU10046.1	172 aa
Sequence 4: AN1761.1	203 aa
Alignment Length: 215 aa
Number Identitical Residues: 109 aa
Alignment Score (without introns) 4999


MG00600.1 	MSS------PRRRIETD0SMTTVSITPKPILTRCV~DIMSPRRDA~AVANSSLS--AV2Q
NCU10046.1	MSS------PRRRIETD0VMK--------------~---------~-----------M2Q
FG09313.1 	MSTGCTDLNSTAQLHTD~KNRLMSDYEVTLVNDNT1TSVCPSSPL~VTSSDSLTDTLR~Q
AN1761.1  	MSS------PKRRIETD0VMKYEHSRLVRLSIPVT~TN--RSTGL1YGLPPLRKGLVK2Q
          	**:      .  ::.**                                    ..    *

MG00600.1 	EFYVVFKGPEET1PFEGGTWKVHVELPDQYPYKSPSIG~FVNRIFHPNIDEL2S--GSVC
NCU10046.1	EFYVRFKGPAET1PFEGGIWKVRVELPDQYPYKSPSIG~FVNRIFHPNIDEL2S--GSVC
FG09313.1 	EFYVRFKGPAET1PFEGGTWKVHVELPDTYPYKSPSIG~FVNRIFHPNIDEL2S--GSVC
AN1761.1  	EFYVRFKGPEET1PFAGGHWKIHVELPDQYPYKSPSIG2YT--VFAPIVDRL~HRSGSVC
          	**** **** ** ** ** **::***** ********* :.  :* * :*.*   :****

MG00600.1 	LDVINQTWSPMFDMINIFEVFLPQLLRYPNPTDPLNGEAAALMLREPKSYEAKVKA1E~Y
NCU10046.1	LDVINQTWSPMFDMINIFEVFLPQLLRYPNPTDPLNGEAAAMLLREPKTYENKVK-~E1Y
FG09313.1 	LDVINQTWSPMFDMINIFEVFLPQLLRYPNPTDPLNGEAAALLIREPKSYDAKVK-~E1Y
AN1761.1  	LDVINQTWSPMYDMINIFEVFLPQLLRYPNPSDPLNGEAAAMLMREPKSYEAKVK-~E1Y
          	***********:*******************:*********:::****:*: ***  * *

MG00600.1 	VQRYASKEAAADAGNESEDDSDMSSVASFGDEDEE-PAGTMDDV
NCU10046.1	VQKYASKDAADEADAESEDDGELSSVASFDEEDDEEAAGKMDDV
FG09313.1 	VQKYASKEAADEAGAESEDDDELSSVASFGDDDDE-PAGQMDDV
AN1761.1  	VAKYASKDAVDDAGEDTESEDELSSAGSYESDGEE-PAGRLDDV
          	* :****:*. :*. ::*.:.::**..*: .:.:* .** :***
```
